# Predicting Candidate Genes Based on Combined Network Topological Features: A Case Study in Coronary Artery Disease

**DOI:** 10.1371/journal.pone.0039542

**Published:** 2012-06-22

**Authors:** Liangcai Zhang, Xu Li, Jingxie Tai, Wan Li, Lina Chen

**Affiliations:** College of Bioinformatics Science and Technology, Harbin Medical University, Harbin, Heilongjiang Province, China; FuWai hospital, Chinese Academy of Medical Sciences, China

## Abstract

Predicting candidate genes using gene expression profiles and unbiased protein-protein interactions (PPI) contributes a lot in deciphering the pathogenesis of complex diseases. Recent studies showed that there are significant disparities in network topological features between non-disease and disease genes in protein-protein interaction settings. Integrated methods could consider their characteristics comprehensively in a biological network. In this study, we introduce a novel computational method, based on combined network topological features, to construct a combined classifier and then use it to predict candidate genes for coronary artery diseases (CAD). As a result, 276 novel candidate genes were predicted and were found to share similar functions to known disease genes. The majority of the candidate genes were cross-validated by other three methods. Our method will be useful in the search for candidate genes of other diseases.

## Introduction

Many complex diseases *like* coronary artery disease result from a complex interplay of multiple genes. A great challenging of biomedical research is to identify candidate genes, which will further help elucidate their roles in the pathogenesis of complex diseases. Recent accumulation of reliable molecular interaction data has boosted progress in the discovery of novel susceptibility genes and fueled expectations about opportunities of computational approaches for distinguishing disease-related genes from non-disease ones. Recent studies on the prediction of candidate genes based on PPI networks alone or in addition to gene expression profiles [1], [2], [3] could return potential candidate genes and facilitate a better understanding of the role of their topological features in the prediction of susceptibility genes so far, but all have investigated only one or two network topological features. Previous discoveries [4], [5], [6], [7], [8] demonstrated that direct interaction partners of a protein are likely to share similar functions with it, and causative genes of some complex disease tends to reside in the same network communities such as biological modules, protein complexes, pathways or subnetworks of a given biological network. Some further graph-theoretical analyses of molecular interaction networks [8], [9], [10], [11] have succeeded in identifying biological network modules and deciphering the association between genes and diseases. To sum up, a unified underlying hypothesis states that genes sharing similar network topological features with known disease genes may result in the same phenotypes. Support Vector Machine (SVM), assumed as ‘a machine-learning algorithm’ based on the Statistical Learning Theory (SLT), is generally introduced into tackling classification problems. SVMs could have good classification effects and performances with a few learning samples [12]. SVMs make predictions and give final classification decisions through learning from existing knowledge automatically [13]. Recently, SVMs have become very popular in the applications of a wide variety of biological questions or topics [13], [14], [15], [16], [17], [18], including gene classification, functional prediction and cancer tissue classifications. To a certain extent, identifying candidate genes for a complex disease could be regarded as a problem of distinguishing disease genes from non-disease genes, which is one of the right problems that SVMs work on. Apart from that, with the accumulation of human protein-protein interaction networks, it is also necessary to introduce novel approaches to find out effective network topological features for gene classifications, and then further aid in the prediction of candidate disease genes.

According to the hypothesis that genes sharing similar network topological features in biological network settings might result in the same or similar phenotypes, we introduced a method, termed eCTFMing, to identify effective combined network topological features and then utilize them into the candidate gene predication. In this article, we firstly identified whether the primary features are effective or not in classification of disease- and non-disease genes, and then screened out effective features from primary features. Finally, a set of optima combined features was constructed to carry out our final prediction. After that, functional coherence between candidate and known disease genes was examined to verify associations of candidate genes with the disease. To evaluate the performance, we compared eCTFMining with three other methods.

## Materials and Methods

**Figure 1:**
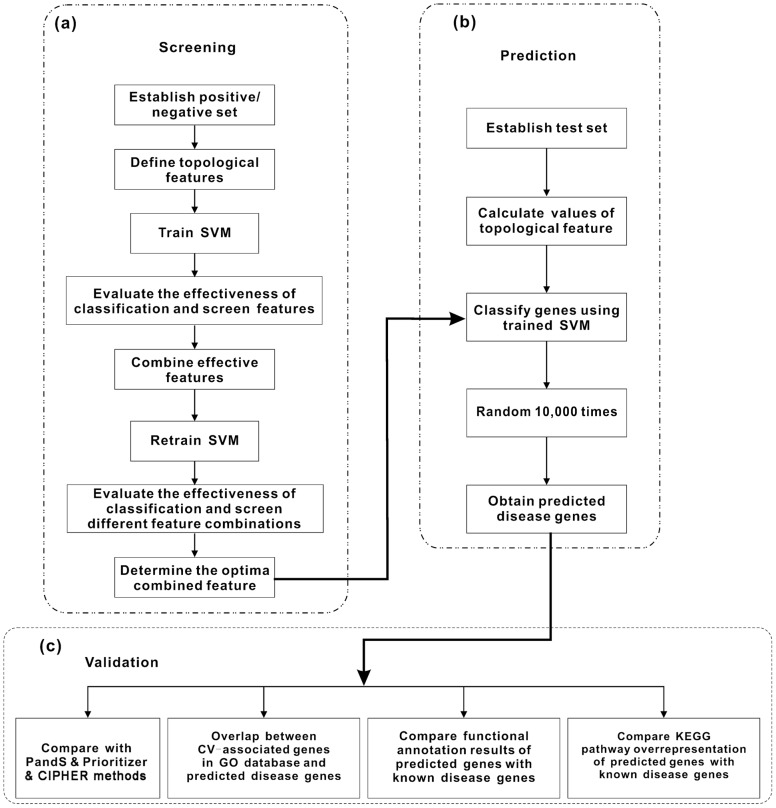
Flowchart of the eCTFMing method. This flowchart contains three mainly steps: (a) Screening of optima combined feature, (b) prediction strategies and (c) validation followed throughout this study.

In this article, we introduced a method, called eCTFMing, to identify candidate disease genes by analyzing network topological features of genes in a PPI network. Figure 1 shows the detailed steps of this method(see in Figure 1).

### Data Sources and Preprocessing

The interactions of the Human Protein Reference Database (HPRD, http://www.hprd.org/) are all manually extracted from literatures by expert biologists who read, interpret and analyze the published data. In order to validate whether our approach relies upon the PPI data, we used an unbiased data sources from Sebastian Kohler *et al.* [19] to predict candidate genes. Sebastian Kohler *et al.* [19] constructed a PPI network which had 258314 interactions between 13725 genes. This PPI network contains five PPI datasets from *Homo Sapiens*, *Mus musculus*, *Drosophila melanogaster*, *Caenorhabditis elegans*, and *Saccharomyces cerevisiae* and these datasets comprise interactions extracted from HPRD, BIND and BioGrid and additional interactions from IntACT, DIP and STRING.

Gene expression profiles of CAD were downloaded from the NCBI Gene Expression Omnibus (GEO, http://www.ncbi.nlm.nih.gov/geo/) database with the accession numbers being GSE974, GSE1145 and GSE2014. During the preprocessing step, differential expression analysis was performed to screen out differentially-expressed genes within each profile. A global median normalization was carried out. The differentially expressed genes were identified by t-test and a p-value cutoff of 0.05 was selected to find differentially expressed genes. An intersection manipulation was carried out to get differentially-expressed genes in common in these three profiles. It should be noted that the chromosomal locations for the differentially-expressed genes were downloaded from the Ensemble database (http://www.ensembl.org/index.html). In addition, one hundred and thirty eight known disease genes and one hundred and sixty eight disease loci for this disease were acquired from the Online Mendelian Inheritance in Man database (OMIM, http://www.ncbi.nlm.nih.gov/sites/entrez?db=OMIM).

### Construction of Positive, Negative and Test Sets

Positive genes were defined as CAD disease genes collected from the OMIM online database and literature mining. Negative genes consisted of all the remaining genes in the human PPI network by excluding positive genes and differentially-expressed genes. The test gene set is the intersection between the differentially-expressed genes and those genes located within the disease loci.

### Selection of Network Topological Features

For all these three gene sets, we defined six commonly-used measurements for each gene, g*_i_*, to evaluate its network topological features in the PPI network.

- Degree (D): in the network, the degree of gene g*_i_* is equal to the number of its adjacent links.
- Neighbor count of disease genes (N): N is the number of neighboring disease genes among all the neighboring genes of gene g*_i_*.
- Ratio of disease genes in neighbor (R): R is the ratio of the count of neighboring disease genes to the count of all neighbor genes.
- Betweenness centrality (B): the betweenness centrality of gene g*_i_* is the count of shortest paths between other nodes that run through the node of interest.
- Clustering coefficient (C): C is the ratio of the number of edges between a vertex’s neighbors to the total possible number of edges between the vertex’s neighbors.
- Mean shortest path length to disease gene (M): A shortest path between two nodes corresponds to the minimum number of edges that have to be traversed in a network to get from one node to the other. In this study, we calculated the average length of shortest paths from gene g*_i_* to all the disease genes.

According to these network topological measurements, we got a vector V of topological features, labeled as (D, N, R, B, C, M), and then tested whether there was a significant disparity between positive and negative gene sets or not. In this step, a Wilcoxon rank sum test analysis for each measurement was performed between positive and negative sets and the corresponding significance threshold (p value) was set to 0.05.

### Identification of Effective Topological Features

A ten-fold cross-validation test was used to evaluate the classification performance and then screen out optima training set for SVM classifications. Six specific topological features were the primary input features for training six support vector machines with each one performing for 1,000 randomizations. The prediction power was evaluated by precision, true positive rate (TPR) and false positive rate (FPR). These three indexes were jointly used to pre-screen these topological features.

### Combination of Effective Features and Identification of Optima Combined Features

It should be noted that at this step all possible combinations were considered. If we have n features in the topological feature vector V, there will be 2^n^ combinations in total. Then we combined the effective topological features, retrained the SVMs using each combined feature out of 2^n^−1 combinations and performed the SVM classification predictions for 1,000 randomizations. After that, the classification performance was evaluated by precision, TPR and FPR. The best combinations were chosen as optima combined features.

### Prediction of Candidate Genes

The optima combined features were used to pick out candidate genes of CAD from the test gene set. This process was performed repeatedly for 10,000 randomizations. With each randomization, we tested whether each gene could be classified to be a disease gene. If so, this gene was finally assumed as a candidate gene in our result.

**Table 1:** Summary of Significance analysis and median values of the topological features for the positive genes and negative genes.

	Mean		p value
	Positive Set	Negative Set	
Degree	6.9805	16.735	5.11E−15
Neighbor number of disease gene	0.1916	1.0427	0
Ratio of disease gene in neighbor	0.0263	0.1115	0
Betweenness centrality	2.41E+04	8.31E+04	1.40E−13
Clustering coefficient	0.1048	0.0882	7.61E−05
Mean shortest path length to disease gene	3.7379	3.2778	2.04E−19

**Figure 2:**
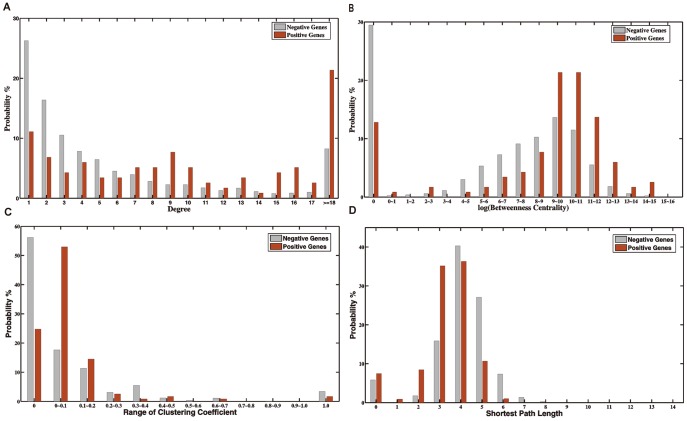
Probability distributions of the ‘Degree’, ‘Betweenness Centrality’, ‘Clustering Coefficient’ and ‘Mean Shortest Path Length’ topological features for positive and negative gene sets.

### Analysis of Functional Coherence

We applied BiNGO [20], a Cytoscape [21] plug-in, to assess which Gene Ontology (GO, http://www.geneontology.org/) terms were significantly overrepresented in a set of known disease genes. Benjamini and Hochberg multiple testing corrections were used to adjust the raw P-values with the significance threshold being 0.05. Meanwhile, GO function annotations were acquired for candidate genes. And then, we tested whether candidate genes shared the same functions with known disease genes to validate the associations of candidate genes with this disease.

The Web-based Gene Set Analysis Toolkit [22] (WebGestalt) is a suite of tools for functional enrichment analysis in various biological contexts. Here, we used it to compare candidate and disease gene lists with genes in KEGG pathway contexts respectively to identify significant pathways which candidate and disease genes located in. A significant level of 0.01 was selected as the cutoff for selecting significantly enriched pathway categories.

**Figure 3:**
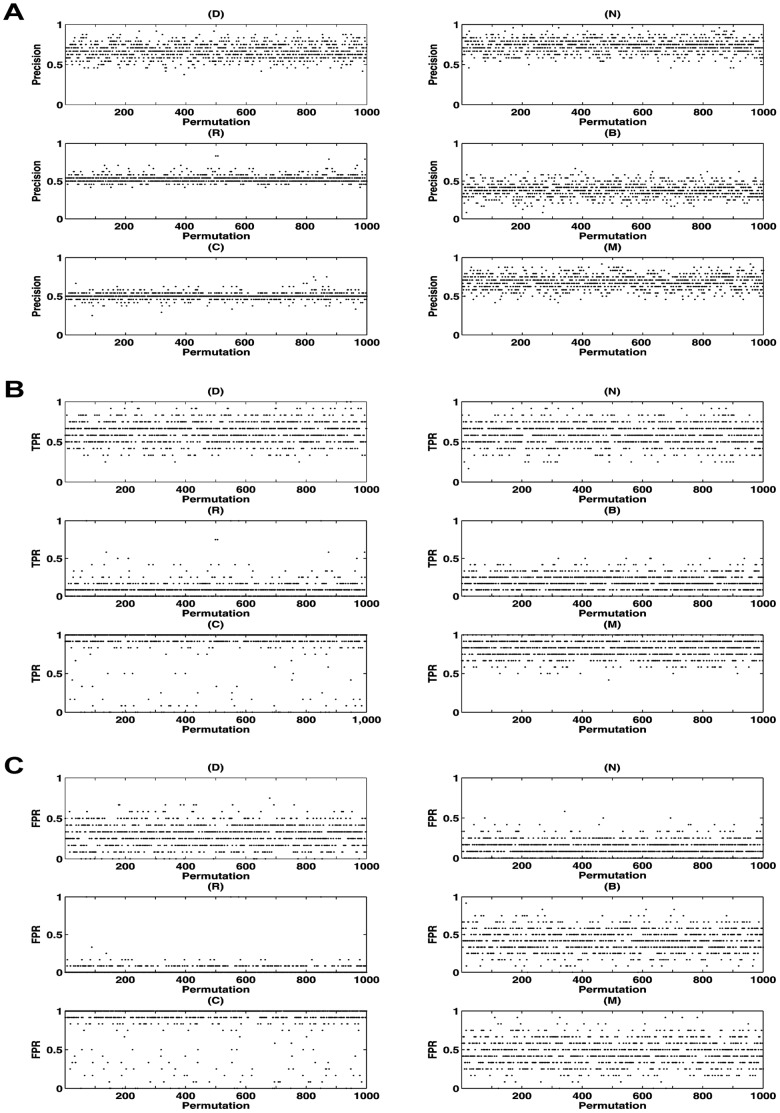
Performances of six topological features by SVM. A: Precision; B: TPR; C: FPR. (D) - Degree; (N) - Neighbor number of disease gene; (R) - Ratio of disease gene in neighbor; (B) - Betweenness centrality; (C) - Clustering coefficient; (M) - Mean shortest path length to disease gene.

## Results

### Selection of Network Topological Features

According to results in Table 1 and Figure 2, we found that these widely-used topological features did have more or less contributions individually for classifications of positive and negative genes. From the probability distribution in Figure 2a, together with median results in Table 1, most CAD positive genes were linked to more interacting neighbors than negative ones in the network. To be more exact, a majority of negative genes had less than eight links to other nodes. In contrast, there were visible differences in their neighboring links between CAD positive and negative genes when the values went higher than 8. Noticeably, most positive genes were connected to more than 18 neighbors, indicating that CAD positive genes, to some degree, are very likely to be hub node members in the network, which is coincident with previous study [23]. In addition, nearly 87.3% of negative genes had no direct interactions with known disease genes, while 32% of positive genes had at least one link to known disease genes. As for the betweenness centrality, positive genes were with higher connectivity to create short path lengths between two nodes across the network than negative genes. These CAD positive genes will naturally experience much higher visits because of this added connectivity (Figure 2b). When it comes to the clustering coefficient, more than 50% of the negative genes had a clustering coefficient of zero, which indicated that the majority of CAD negative genes were likely to be isolated nodes (Figure 2c). Additionally, disease genes had slightly shorter path lengths than non-disease genes, and shortest path lengths were ranging from two to four between disease genes (Figure 2d). Therefore, it seems to be possible to utilize these network topological features to train Support Vector Machines in distinguishing CAD disease genes from non-disease genes through network topological analysis.

**Figure 4:**
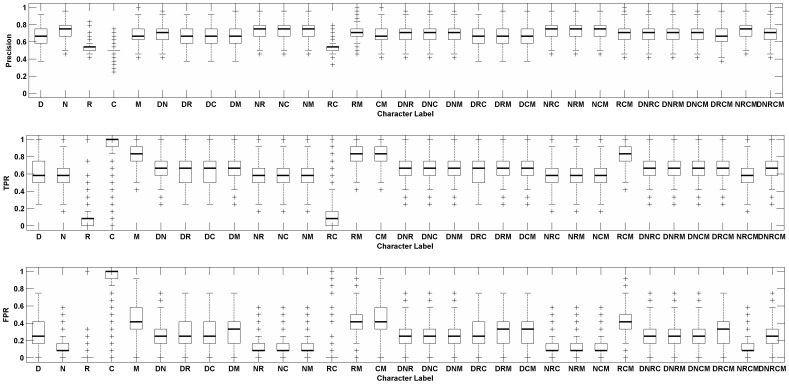
Performances of combined topological features by SVMs. D - Degree; N - Neighbor number of disease gene; R - Ratio of disease gene in neighbor; C - Clustering coefficient; M - Mean shortest path length to disease gene. The character labels represented the combined topological features, respectively.

### Confirmation of Effective Topological Features

As for each of the topological features, a corresponding SVM classifier was trained and its precision, true positive rate (TPR) and false positive rate (FPR) were evaluated to confirm whether this feature was effective in gene classification. One criterion was that each of the resulting features we chose should have relative higher values of classification precision and TPR but lower value of classification FPR. In figure 3, we found the classification performance of the betweenness centrality measurement was of lower values of precision and TPR but higher value of FPR, indicating that betweenness could not be selected as an effective feature in further classification. And the classifiers of D, N and M features had higher precision, respectively. We also found that there were lower FPR using N and R features to train SVM and the FPR of B and C features were both much higher than the others. Afterwards, according to the threshold of precision, TPR and FPR, five network topological features (C, D, M, N, and R) were finally retained and confirmed as effective features for distinguishing CAD disease genes from non-disease genes.

### Combining Topological Features and Screening Optima Combined Features by SVM

We retrained 31 (2^5^−1) SVMs for gene classification with each of the corresponding combinations of five effective topological features as feature inputs. Then their classification performances were evaluated according to values of precision, TPR and FPR (see in Figure 4). From the results, the combined features of N, R, C and M could effectively distinguish disease from non-disease genes. We defined these combined features as optima combined features.

### Disease Genes Prediction

During the SVMs trainings and predictions, we randomly selected negative genes with an identical number of positive genes from the negative gene set as the size of negative gene set was much larger than positive one. For fear of the possible selection bias, this step of disease gene prediction was performed for 10,000 times to get CAD candidate genes using the optima combined features, while the negative gene inputs were different between every two manipulations. After 10,000 predictions, the intersection of each prediction was defined as our final prediction, and 276 candidate genes were finally returned.

**Figure 5:**
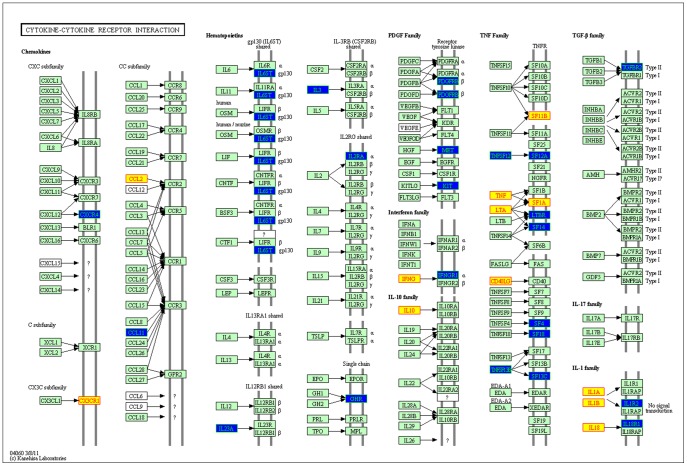
The detailed interaction information of known and candidate disease genes in the KEGG pathway of cytokine-cytokine receptor interaction. Blue nodes represent candidate disease genes, and the yellow ones represent known disease genes.

### Analysis of Functional Coherence

Functional coherence between candidate and known disease genes was examined to verify associations of candidate genes with the disease. In this step, we performed function and pathway enrichment analyses for candidate and disease genes, respectively.

BiNGO, a Cytoscape plugin to assess overrepresentation of Gene Ontology categories in Biological Networks, was used to map the predominant functional themes of the tested gene set on the GO hierarchy, and take advantage of Cytoscape’s versatile visualization environment to produce an intuitive and customizable visual representation of the results. Genes associated with the same disease phenotype were found to share common cellular and functional characteristics, as annotated in the Gene Ontology (See in Figure S1 and Table S1). We found that a majority of CAD candidate genes that had similar network topological features tended to have a significantly functional relatedness to known disease genes in following categories such as *protein binding, receptor binding, molecular transducer activity, signal transducer activity, receptor activity, oxidoreductase activity*, *hydroxymethylglutaryl-CoA reductase (NADPH) activity* and so on. Moreover, a partial of CAD candidate and known disease genes were annotated on the GO terms of ‘*auxiliary transport protein activity’, ‘carbohydrate binding’* and ‘*lipid binding’*. Genes with similar phenotypes might share similar functions; therefore we compared the 276 candidate genes with the cardiovascular GO annotation initiative genes (Table S1). We found that 216 of the candidate genes were in the Cardiovascular GO Annotation list of genes known to be associated with cardiovascular processes. For example, one gene (CD55) was found to influence monocyte cholesterol homeostasis and participate in the development of CAD [24].

**Figure 6:**
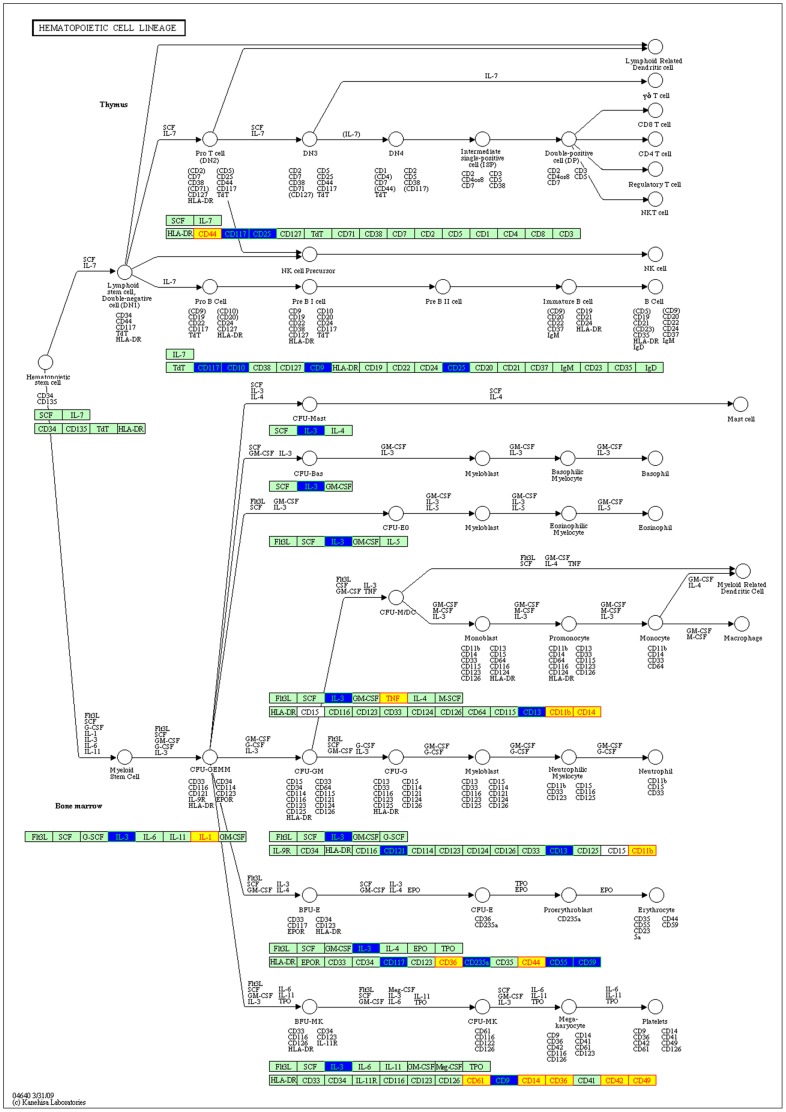
The detailed interaction information of known and candidate disease genes in the KEGG pathway of hematopoietic cell lineage. Blue nodes represent candidate disease genes, and the yellow ones represent known disease genes.

WebGestalt, an online analysis platform, is a suite of tools for functional enrichment analysis in various biological contexts. It was used to evaluate the overlaps of candidate and disease gene lists with genes in KEGG pathway contexts respectively and then significant pathways which candidate and disease genes located in were identified. We found many candidate genes participated in specific KEGG pathways where known disease genes were over-represented and these two gene sets shared 60 (nearly 25%) common KEGG pathways such as *cytokine-cytokine receptor interaction*, *Regulation of actin cytoskeleton*, *Focal adhesion*, *MAPK signaling pathway*, *Adipocytokine receptor signaling pathway*, *VEGF signaling pathway*, *Type I diabetes mellitus*, *hematopoietic cell lineage*, and *complement and coagulation cascades* and so on (Table S2).

One interesting result we found was that known and candidate disease genes were over-represented in the KEGG pathway of cytokine-cytokine receptor interaction (Figure 5), which might suggest involvement of inflammation in the CAD process. In this pathway, candidate genes like LTBR, IFNGR1, SF14 and IL1R2 tended to have direct interactions with known disease genes. Some of our results had been cross-validated by previous studies. It is known that the cytokine network is a complex and dynamic system composed of numerous biological responses in human body. Cytokines and their interaction with the coagulation system play important roles, especially [25] in the maintenance of the thrombo-hemorrhagic balance *in vivo* in human subjects. Martins *et al.* [26] declared that cytokine profiles may have a role in differentiating patients with CAD with myocardial infarction from those with chest pain due to other disorders and in deciphering the role of inflammation in the pathogenesis of CAD. Tiroch *et al.* [27] argued that gene expression analysis in atherectomy specimens derived from restenotic coronary lesions indicated activation of Interferon gamma signaling in neointimal smooth muscle cells, which, in some way, verified the role of the candidate gene, the Interferon gamma (IFNG) gene, in the pathogenesis of CAD. Kim *et al.* [28] further suggested IFNG gene may be one of the factors determining the extent of CAD in the Korean population. Also, ghrelin encoded by the GHR gene, a novel endogenous ligand for the growth hormone secretagogue receptor, was considered [29] to exert a protective effect against atherosclerosis. The LEPR gene, a member of the family of cytokine receptors type I, encodes the protein of leptin, which is also an independent risk factor [30], [31] for CAD. Because thrombus formation is a major cause of acute coronary events and leptin was shown previously to facilitate ADP-induced platelet aggregation. Leptin-induced platelet activity required activation of a signaling cascade that included the long form of the leptin receptor, three kinases of JAK2, PI3K and PKB/Akt, IRS-1, and PDE3A. In addition, smooth muscle cell (SMC) proliferation in atherosclerosis was found [32] to mediate through the interaction of growth factors like platelet-derived growth factor-RB (PDGF-RB) and insulin-like growth factor-1 (IGF-1) and their receptors (R).

**Figure 7:**
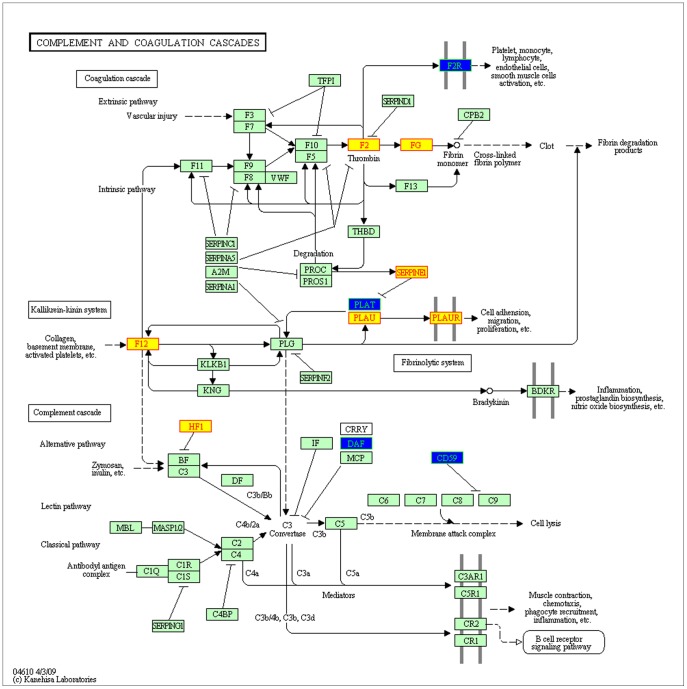
The detailed interaction information of known and candidate disease genes in the KEGG pathway of complement and coagulation cascades. Blue nodes represent candidate disease genes, and the yellow ones represent known disease genes.

Another interesting findings was that known and candidate disease genes were over-represented in the KEGG pathway of hematopoietic cell lineage (Figure 6), which might also suggest involvement of inflammation in the CAD development. In the pathway, IL2RA, IL3, CD9, CD10, CD25 and CD117 appeared to interact with known disease genes directly, while some of our results were verified by previous CAD-related studies. Tabata *et al.* [33] declared that engagement of one candidate gene, namely the cell-adhesion CD44 gene, with low molecular weight hyaluronan was centrally involved in the inflammatory pathogenesis of athelosclerotic plaques through migration of monocytes and foamed macrophage differentiation. Hagg *et al.* [34] showed that pro-inflammatory cytokines could affect the expression of CD44 and also examined the role of elevated CD44 expression levels in human macrophages. To be more exact, an IL-6-CD44 feedback loop was found in macrophages and this positive feedback loop may be the cause of aggravating atherosclerosis development. Wolf *et al.* [24] found that differential CD14-dependent receptor clustering within microdomains experienced noticeable effects in response to *in vivo* lipopolysaccharide (LPS) and/or atherogenic lipoprotein activation. Moreover, they observed more evidences of increased DRM-association of the GPI-anchored proteins CD14, CD55, CD64, the scavenger receptors CD36, CD91 and CD163, the integrin CD11a, and complement receptor 3 complex CD11b/CD18 from patients with CAD.

In figure 7, we assumed the KEGG pathway of complement and coagulation cascades might be CAD risk related. Candidate and known disease genes in this pathway, appearing to be altered in patients with CAD, acted an important role [35] in the complement cascade and the innate immunity as well as in the regulation of proteolytic cascade involved in inflammatory and coagulation processes. Apart from that, the increased levels or genetic mutations in the F2 receptor (F2R) involved in coagulation may play a role in the pathogenesis of coronary artery disease. By virtue of its strong correlation to plasma TNF-alpha, F2R may be an important mediator [36] of the effects of inflammation on the vessel wall, and this study further provided promising strategies of blocking F2R to the treatment of human atherosclerosis. Another study demonstrated that in men F2R genetic variants tended to influence the risk of the occurrence of myocardial infarction mainly through an interaction with IL6 serum levels. Changes in plasminogen activator inhibitor 1 and tissue-type plasminogen activator (PLAT gene) [37] were detected during exercise in patients with coronary artery disease. Karlsson *et al.* [38] argued that there might be a role of plasma and tissue-type plasminogen activator in coronary atherosclerosis, suggesting that treatment with recombinant tissue-type plasminogen activator (PLAT), in unstable coronary artery disease in men reduced myocardial ischemia.

### Comparison with other Methods

Similar to our method, Prioritizer and PandS methods could be used to rank genes related to a specific disorder with the assumption that disease genes in a specific disorder are usually functionally related. Also, on the basis of the assumption that phenotypically similar diseases are caused by functionally related genes, CIPHER [6] could integrate human protein–protein interactions, disease phenotype similarities, and known gene–phenotype associations effectively, assuming as a global network-based inference approach for human disease gene identification. While, Prioritizer [39] ranks genes based on their functional interactions with genes in different susceptibility loci. As for PandS(PROSPECTR and SUSPECTS combined) [40], PROSPECTS differentiates between disease and non-disease genes using sequence-based features; SUSPECTS scores candidate genes using the PROSPECTR algorithm and also assess the similarity between their annotations and those of known disease genes. To validate the results with those obtained by our method, the Prioritizer, PandS and CIPHER methods were introduced to explore CAD candidate genes through the genome-wide scan of CAD susceptibility loci. From the results, we can safely draw a conclusion that our candidate CAD genes could be cross-validated by these well-known disease gene identification methods and show close associations in shared disease risk pathways or GO functional categories with known CAD disease genes (see in Table S1).

## Discussion

Coronary artery disease, as one of complex diseases, is assumed to be caused by the combined effects of multiple disease genes. We found that the eCTFMining method could take into account of different network topological features of genes in the biological network to characterize their possible functional relationships with known disease genes and further aid in the disease gene identification. In our research, CAD-related genes are likely to have the following characteristics: *i*) they tend to be hubs in the network, often with more links to other genes than non-disease genes; *ii*) these genes follow the rule of ‘guilty by association’, and if there are more disease-related interacting neighbors for a gene, this gene is more likely to be a candidate; *iii*) the neighborhood of one disease gene is well connected in the network namely that CAD disease genes have much higher clustering coefficient values than non-disease genes; and *iv*) disease genes are likely to locate in small-world subnetworks as the mean shortest path length between disease genes is generally less than 4 (see in Figure S2).

It must be noted that several human processes like the cytokine-cytokine receptor interaction pathway, the hematopoietic cell lineage pathway, the complement and coagulation cascades pathway show significant associations with coronary artery disease, which also suggest involvement of inflammation in the disease development. Thus, there might be common mechanisms between the inflammatory pathogenesis and coronary artery disease. Apart from that, some attentions should be paid to those pathways as they mainly consist of CAD candidate genes and known disease genes. Here, we declare that studies on CAD disease-related pathways might provide insights into possibly promising drug target discovery.

Different from other methods based on single topological feature, our method takes advantage of all the commonly-used network topological features and then searches the optima feature combinations for CAD disease gene identification. Our method has returned affluent results in CAD disease gene prediction and a majority of them have been cross-validated by another method or two. Few candidate genes that are not verified by current knowledge systems would help researchers to create new hypothesis for experiments. However, our method relies partly on the confidence in, and quality of, PPI or known disease gene datasets. To sum up, with further improvement of protein-protein interaction networks and disease genes databases, the performance of our method could be more effective and dependable.

## Supporting Information

Figure S1 **The GO function annotation of known disease genes and candidate disease genes.** Dark yellow circular nodes are more significantly overrepresented by known disease genes. White nodes are not significantly overrepresented; they are included to show the yellow nodes in the context of the GO hierarchy. The area of a node is proportional to the number of genes in the test set annotated to the corresponding GO category. The square nodes are significantly overrepresented by known and candidate disease genes.
(TIF)

Figure S2 **Distribution of the topological features of positive/negative genes and candidate genes.**
(TIF)

Table S1 **List of 276 candidate disease genes.**
(DOC)

Table S2 **Pathway list of enrichment by known and candidate disease genes.**
(DOC)
