# Supplementary figures and images for: Predicting Candidate Genes Based on Combined Network Topological Features: A Case Study in Coronary Artery Disease
Source: PLoS One. 2012 Jun 22;7(6):e39542. doi: 10.1371/journal.pone.0039542 (PMC3382204; doi:10.1371/journal.pone.0039542)

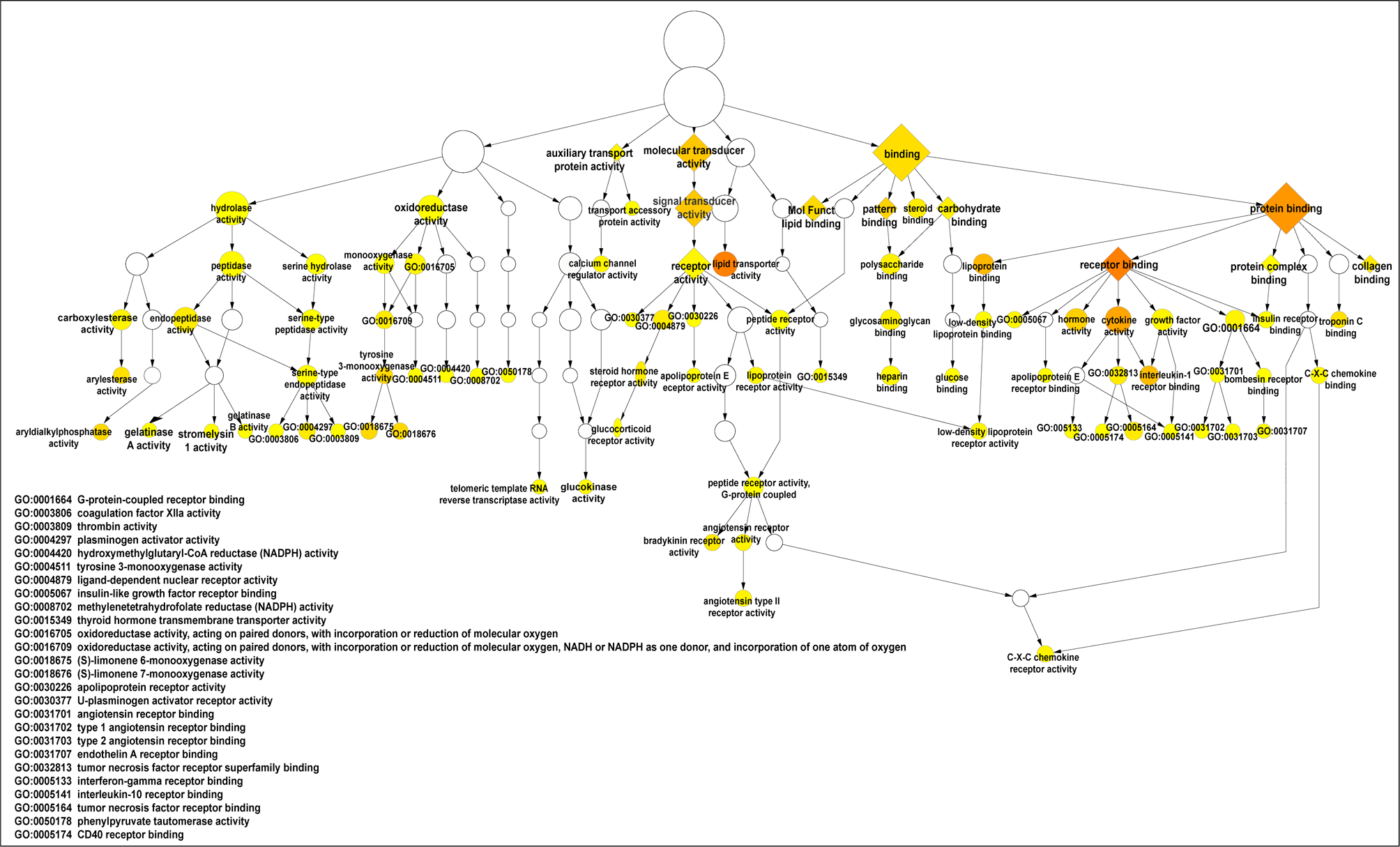

Supplement: Figure S1 — The GO function annotation of known disease genes and candidate disease genes. Dark yellow circular nodes are more significantly overrepresented by known disease genes. White nodes are not significantly overrepresented; they are included to show the yellow nodes in the context of the GO hierarchy. The area of a node is proportional to the number of genes in the test set annotated to the corresponding GO category. The square nodes are significantly overrepresented by known and candidate disease genes. (TIF) [file pone.0039542.s001.tif]

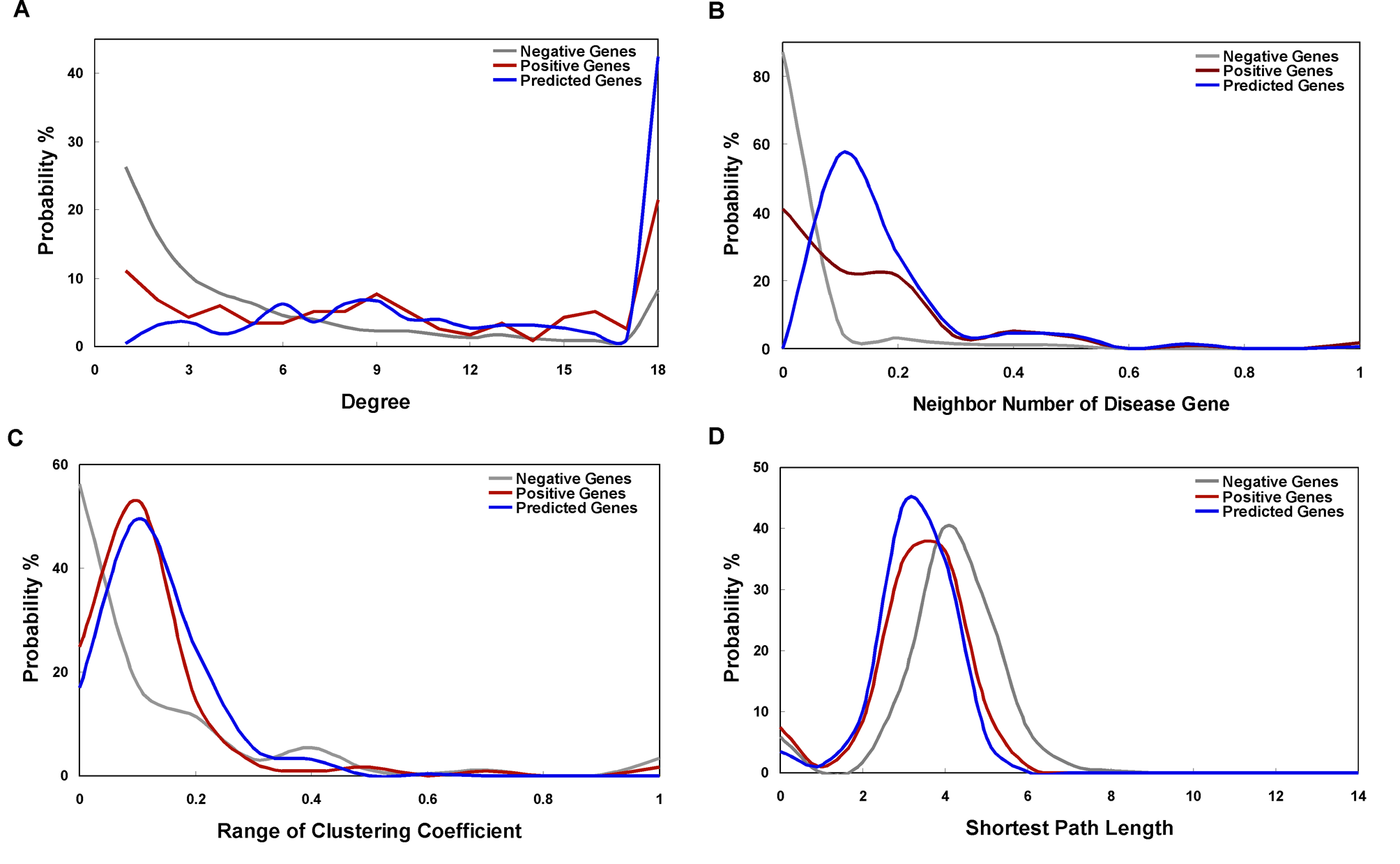

Supplement: Figure S2 — Distribution of the topological features of positive/negative genes and candidate genes. (TIF) [file pone.0039542.s002.tif]
