# Supplementary material for: Predicting Candidate Genes Based on Combined Network Topological Features: A Case Study in Coronary Artery Disease
Source: PLoS One. 2012 Jun 22;7(6):e39542. doi: 10.1371/journal.pone.0039542 (PMC3382204; doi:10.1371/journal.pone.0039542)
Supplement: Table S1 — List of 276 candidate disease genes. (DOC) [file pone.0039542.s003.doc]

**Table S1. List of 276 candidate disease genes.**

| ***Gene ID*** | ***Gene Symbol*** | ***Other methods*** | | | ***Cardiovascular GO Annotation Initiative Gene List*** | ***GO Function Annotation*** | ***Pathway*** |
| --- | --- | --- | --- | --- | --- | --- | --- |
|  |  | **Prioritizer** | **PandS** | **CIPHER** |  |  |  |
| 25 | ABL1 |  | • |  |  | * | * |
| 52 | ACP1 |  | • |  | - | * | * |
| 58 | ACTA1 |  | • | • | - | * | * |
| 60 | ACTB |  | • | • |  |  | * |
| 70 | ACTC1 |  | • | • | - |  |  |
| 156 | ADRBK1 | • | • |  | - | * | * |
| 290 | ANPEP | • | • |  | - |  | * |
| 322 | APBB1 | • | • |  |  |  | * |
| 330 | BIRC3 | • |  |  | - |  | * |
| 350 | APOH |  | • |  | - |  |  |
| 351 | APP | • | • |  | - |  | * |
| ***367*** | ***AR*** | • | • | • | - | * | * |
| 567 | B2M | • |  | • | - |  | * |
| 598 | BCL2L1 | • |  |  | - | * | * |
| 602 | BCL3 | • |  |  | - | * | * |
| 633 | BGN |  |  | • | - | * | * |
| 652 | BMP4 | • | • |  | - |  | * |
| 667 | DST | • | • |  | - |  |  |
| 672 | BRCA1 | • | • |  | - | * | * |
| 811 | CALR | • | • | • | - |  | * |
| 821 | CANX | • | • | • |  |  | * |
| 826 | CAPNS1 | • | • |  | - |  |  |
| 847 | CAT | • | • |  | - |  | * |
| 857 | CAV1 | • |  |  | - |  | * |
| 928 | CD9 | • | • |  |  | * | * |
| ***929*** | ***CD14*** | • | • | • | - | * | * |
| ***960*** | ***CD44*** | • | • | • | - | * | * |
| 965 | CD58 | • | • |  |  |  | * |
| 966 | CD59 | • | • |  |  |  | * |
| 967 | CD63 | • |  |  |  |  |  |
| 969 | CD69 | • |  |  |  |  |  |
| 1000 | CDH2 | • |  |  | - | * | * |
| 1017 | CDK2 | • | • |  | - |  |  |
| 1027 | CDKN1B |  | • |  | - |  | * |
| 1116 | CHI3L1 | • | • |  | - |  |  |
| 1154 | CISH | • |  |  | - |  | * |
| 1278 | COL1A2 | • | • |  | - |  | * |
| 1281 | COL3A1 | • | • |  | - |  | * |
| 1287 | COL4A5 | • | • |  | - | * | * |
| 1288 | COL4A6 | • |  |  | - | * | * |
| 1312 | COMT |  | • |  | - |  | * |
| 1399 | CRKL | • | • |  | - | * | * |
| 1404 | HAPLN1 | • | • |  | - | * | * |
| 1482 | NKX2-5 | • | • | • | - |  |  |
| 1490 | CTGF | • | • |  | - |  |  |
| 1508 | CTSB | • |  |  | - | * | * |
| 1514 | CTSL |  |  |  |  |  | * |
| 1593 | CYP27A1 |  | • |  | - | * | * |
| 1604 | CD55 |  | • |  |  | * | * |
| 1616 | DAXX | • |  |  |  | * | * |
| 1813 | DRD2 |  | • |  | - |  | * |
| 1854 | DUT |  | • | • | - |  |  |
| 1889 | ECE1 |  | • |  | - | * | * |
| 1906 | EDN1 |  | • |  | - | * |  |
| 1909 | EDNRA |  | • |  | - | * | * |
| 2022 | ENG |  |  | • | - |  |  |
| 2052 | EPHX1 |  | • | • | - |  |  |
| 2053 | EPHX2 | • | • |  | - |  |  |
| 2064 | ERBB2 | • | • |  | - | * | * |
| 2098 | ESD | • | • |  |  |  |  |
| ***2099*** | ***ESR1*** | ***•*** | ***•*** | • | ***-*** | ******* | ******* |
| 2149 | F2R | • | • | • | - | * | * |
| 2170 | FABP3 |  | • | • | - |  | * |
| 2180 | ACSL1 |  | • |  | - |  | * |
| 2217 | FCGRT |  |  | • |  |  |  |
| 2268 | FGR |  | • |  |  |  |  |
| 2317 | FLNB | • |  |  | - | * | * |
| 2395 | FXN | • | • |  | - |  |  |
| 2475 | FRAP1 | • | • |  |  | * | * |
| 2494 | NR5A2 |  | • |  | - | * | * |
| 2526 | FUT4 | • |  |  |  |  |  |
| 2627 | GATA6 |  |  |  | - |  |  |
| 2690 | GHR | • |  | • | - | * | * |
| 2770 | GNAI1 |  |  | • | - |  | * |
| 2776 | GNAQ |  | • |  | - |  | * |
| 2782 | GNB1 | • | • |  | - | * | * |
| ***2784*** | ***GNB3*** | ***•*** | ***•*** | • | ***-*** |  |  |
| 2821 | GPI |  | • |  |  |  | * |
| 2885 | GRB2 |  |  |  | - |  | * |
| 2932 | GSK3B | • | • |  | - | * | * |
| 2950 | GSTP1 |  | • |  | - |  | * |
| 2959 | GTF2B | • | • |  |  | * | * |
| 2993 | GYPA |  | • |  |  |  | * |
| 3001 | GZMA |  | • |  |  |  | * |
| 3065 | HDAC1 | • |  |  |  | * | * |
| 3069 | HDLBP |  | • |  | - |  |  |
| 3091 | HIF1A | • | • |  | - |  | * |
| 3172 | HNF4A | • |  | • | - | * | * |
| 3251 | HPRT1 |  | • |  |  |  | * |
| 3308 | HSPA4 | • |  | • |  | * | * |
| 3309 | HSPA5 |  | • | • |  | * | * |
| 3312 | HSPA8 | • | • | • | - | * | * |
| 3315 | HSPB1 | • |  |  |  |  | * |
| 3320 | HSP90AA1 | • | • | • | - |  | * |
| 3326 | HSP90AB1 |  | • | • | - | * | * |
| 3329 | HSPD1 | • | • |  | - |  | * |
| 3371 | TNC | • |  |  | - |  | * |
| 3416 | IDE | • | • | • | - |  | * |
| 3429 | IFI27 |  |  |  |  |  |  |
| 3459 | IFNGR1 | • | • |  | - | * | * |
| 3479 | IGF1 |  | • |  | - |  | * |
| 3480 | IGF1R |  | • | • | - |  | * |
| 3481 | IGF2 | • | • | • | - |  | * |
| 3482 | IGF2R | • |  |  |  | * | * |
| 3552 | IL1A | • | • |  | - |  | * |
| 3559 | IL2RA |  | • |  | - | * | * |
| 3562 | IL3 |  | • |  | - |  | * |
| 3572 | IL6ST | • | • |  | - | * | * |
| 3611 | ILK | • | • |  |  | * | * |
| 3654 | IRAK1 |  | • |  | - | * | * |
| 3655 | ITGA6 |  | • |  | - |  | * |
| ***3667*** | ***IRS1*** |  |  | • | ***-*** | ******* | ******* |
| 3675 | ITGA3 |  | • |  |  |  | * |
| 3678 | ITGA5 | • | • |  |  |  | * |
| 3685 | ITGAV | • | • |  | - |  | * |
| ***3690*** | ***ITGB3*** | • | ***•*** | • | ***-*** | ******* | ******* |
| 3718 | JAK3 | • | • |  | - |  | * |
| 3815 | KIT |  |  |  | - |  | * |
| 3856 | KRT8 | • |  |  |  |  |  |
| 3898 | LAD1 | • |  |  | - |  |  |
| 3929 | LBP | • | • | • | - | * | * |
| 4000 | LMNA |  |  | • | - |  |  |
| ***4023*** | ***LPL*** | ***•*** | ***•*** | • | ***-*** | ******* | ******* |
| 4043 | LRPAP1 |  | • | • | - |  |  |
| ***4049*** | ***LTA*** | ***•*** | ***•*** | • | ***-*** | ******* | ******* |
| 4055 | LTBR | • |  |  |  | * | * |
| 4074 | M6PR | • |  |  |  |  |  |
| 4088 | SMAD3 |  | • | • | - |  | * |
| 4145 | MATK | • | • |  |  | * | * |
| 4210 | MEFV | • | • |  | - |  |  |
| 4214 | MAP3K1 | • | • | • | - |  | * |
| 4221 | MEN1 | • | • |  | - | * | * |
| 4233 | MET | • |  |  | - |  | * |
| 4282 | MIF | • | • |  | - | * | * |
| 4311 | MME |  | • |  | - |  | * |
| 4322 | MMP13 | • | • |  | - | * | * |
| 4478 | MSN |  | • |  |  |  | * |
| 4524 | MTHFR | • | • | • | - |  |  |
| 4607 | MYBPC3 | • | • | • | - |  |  |
| 4609 | MYC |  | • |  | - |  | * |
| 4627 | MYH9 |  | • |  | - |  | * |
| 4633 | MYL2 | • | • | • | - |  | * |
| 4659 | PPP1R12A | • | • | • | - |  | * |
| 4772 | NFATC1 |  | • | • | - |  | * |
| 4791 | NFKB2 | • |  | • | - | * | * |
| 4793 | NFKBIB | • |  | • | - | * | * |
| 4794 | NFKBIE | • |  |  |  | * | * |
| 4803 | NGFB |  | • |  |  | * | * |
| ***4846*** | ***NOS3*** | ***•*** | • | • | ***-*** | ******* | ******* |
| 4856 | NOV | • | • |  |  | * | * |
| 5034 | P4HB | • | • |  | - | * |  |
| 5045 | FURIN | • |  | • | - |  |  |
| ***5054*** | ***SERPINE1*** |  | • | • | ***-*** | ******* | ******* |
| 5106 | PCK2 |  | • |  | - |  | * |
| 5133 | PDCD1 | • | • |  | - |  | * |
| 5159 | PDGFRB | • |  | • | - | * | * |
| 5168 | ENPP2 |  | • |  |  |  |  |
| 5170 | PDPK1 | • |  |  | - | * | * |
| 5229 | PGGT1B | • | • |  |  |  |  |
| 5236 | PGM1 | • | • | • |  |  | * |
| 5238 | PGM3 | • | • | • |  |  |  |
| 5295 | PIK3R1 | • | • | • | - |  | * |
| 5327 | PLAT | • | • |  | - | * | * |
| 5328 | PLAU | • | • |  | - |  | * |
| ***5329*** | ***PLAUR*** | ***•*** | • | • | ***-*** | ******* | ******* |
| 5338 | PLD2 | • | • |  | - |  | * |
| 5467 | PPARD | • | • |  | - | * | * |
| 5469 | PPARBP | • | • |  |  | * | * |
| 5506 | PPP1R3A | • | • | • | - |  | * |
| 5581 | PRKCE |  | • |  | - |  | * |
| 5585 | PKN1 | • |  |  | - |  |  |
| 5588 | PRKCQ | • | • | • | - | * | * |
| 5602 | MAPK10 |  |  |  | - |  | * |
| 5604 | MAP2K1 |  | • |  | - |  | * |
| 5605 | MAP2K2 |  |  |  | - |  | * |
| 5606 | MAP2K3 |  |  |  | - |  | * |
| 5610 | EIF2AK2 |  | • | • | - |  |  |
| 5621 | PRNP |  |  |  | - |  | * |
| 5664 | PSEN2 |  | • |  | - | * | * |
| 5914 | RARA | • | • |  | - | * | * |
| 5925 | RB1 | • | • |  | - |  | * |
| 5970 | RELA | • | • |  | - |  | * |
| 5971 | RELB | • |  |  | - | * | * |
| 6014 | RIT2 | • | • |  |  |  |  |
| 6256 | RXRA | • | • |  | - | * | * |
| 6280 | S100A9 |  | • |  | - |  |  |
| ***6347*** | ***CCL2*** | ***•*** | • | • | ***-*** | ******* | ******* |
| 6356 | CCL11 | • | • |  | - | * | * |
| ***6403*** | ***SELP*** |  | • | • | ***-*** | ******* | ******* |
| 6443 | SGCB | • |  | • | - |  |  |
| 6464 | SHC1 |  | • | • | - | * | * |
| 6556 | SLC11A1 | • |  |  |  |  |  |
| 6595 | SMARCA2 | • | • |  |  |  |  |
| 6667 | SP1 | • | • |  | - |  | * |
| 6722 | SRF |  | • | • | - | * | * |
| 6772 | STAT1 |  | • |  | - |  | * |
| 6927 | TCF1 | • | • | • |  | * | * |
| 6928 | TCF2 | • |  | • |  |  | * |
| 6936 | C2orf3 | • |  |  |  |  |  |
| 7020 | TFAP2A | • | • |  |  | * | * |
| 7048 | TGFBR2 | • | • |  | - |  | * |
| 7052 | TGM2 | • |  |  | - | * | * |
| 7058 | THBS2 |  | • |  | - | * | * |
| 7076 | TIMP1 | • | • |  | - |  |  |
| 7078 | TIMP3 | • |  |  | - | * | * |
| 7097 | TLR2 |  | • |  | - | * | * |
| 7098 | TLR3 |  | • |  | - | * | * |
| 7100 | TLR5 | • | • |  | - |  | * |
| ***7132*** | ***TNFRSF1A*** | ***•*** | • | • | ***-*** | ******* | ******* |
| ***7137*** | ***TNNI3*** | ***•*** | • | • | ***-*** | ******* | ******* |
| 7249 | TSC2 | • |  |  |  | * | * |
| 7277 | TUBA1 |  | • |  |  |  | * |
| 7293 | TNFRSF4 | • | • | • | - |  | * |
| 7376 | NR1H2 | • | • |  | - | * |  |
| 7392 | USF2 | • |  | • |  | * | * |
| 7430 | VIL2 | • |  | • |  | * | * |
| 7436 | VLDLR | • | • | • | - |  |  |
| 7850 | IL1R2 |  | • |  | - |  | * |
| 7852 | CXCR4 | • | • |  | - |  | * |
| 8061 | FOSL1 | • | • | • | - | * | * |
| 8087 | FXR1 |  |  |  | - |  |  |
| 8313 | AXIN2 | • |  |  | - |  | * |
| 8439 | NSMAF | • | • |  | - | * | * |
| 8503 | PIK3R3 | • |  | • |  | * | * |
| 8611 | PPAP2A |  | • |  | - |  | * |
| 8648 | NCOA1 |  |  | • | - |  |  |
| 8737 | RIPK1 |  |  |  | - | * | * |
| 8738 | CRADD |  |  |  | - |  |  |
| 8742 | TNFSF12 | • | • | • | - | * | * |
| 8764 | TNFRSF14 |  | • | • |  | * | * |
| 8772 | FADD | • | • | • | - | * | * |
| 8784 | TNFRSF18 | • | • | • |  |  | * |
| 8809 | IL18R1 |  | • | • | - |  | * |
| 8835 | SOCS2 |  | • | • | - |  | * |
| 8841 | HDAC3 | • |  |  | - | * | * |
| 8850 | PCAF |  | • |  |  |  |  |
| 9021 | SOCS3 |  |  | • | - |  | * |
| 9197 | SLC33A1 |  | • |  |  |  |  |
| 9332 | CD163 |  | • |  | - |  |  |
| 9479 | MAPK8IP1 |  |  | • |  |  | * |
| 9611 | NCOR1 |  | • |  | - |  |  |
| 9619 | ABCG1 |  | • |  | - |  |  |
| 10135 | PBEF1 |  | • |  | - |  |  |
| 10403 | KNTC2 |  |  |  |  |  |  |
| 10499 | NCOA2 |  |  |  | - |  |  |
| 10555 | AGPAT2 |  |  |  | - |  | * |
| 10561 | IFI44 |  |  |  |  |  |  |
| 10606 | PAICS |  | • |  |  |  | * |
| 10673 | TNFSF13B |  | • | • |  |  | * |
| 10858 | CYP46A1 |  | • |  | - |  | * |
| 10875 | FGL2 |  | • |  |  |  |  |
| 10987 | COPS5 | • | • |  | - | * | * |
| 10999 | SLC27A4 |  | • | • | - |  | * |
| 11132 | CAPN10 |  | • | • | - |  |  |
| 11162 | NUDT6 |  | • |  |  |  |  |
| 11343 | MGLL |  | • |  | - |  | * |
| 22796 | COG2 |  |  |  |  |  |  |
| 22900 | CARD8 |  |  |  |  |  |  |
| 22937 | SCAP | • | • |  | - |  |  |
| 23118 | MAP3K7IP2 |  |  |  |  |  | * |
| 23533 | PIK3R5 |  |  |  |  |  | * |
| 26040 | SETBP1 |  |  |  |  |  |  |
| 50507 | NOX4 |  | • |  | - |  |  |
| 51083 | GAL |  | • |  | - |  |  |
| 51284 | TLR7 |  | • |  | - |  | * |
| 51330 | TNFRSF12A | • | • | • |  | * | * |
| 51561 | IL23A |  |  |  | - |  | * |
| 54205 | CYCS |  | • |  | - |  | * |
| 54210 | TREM1 |  | • |  | - |  |  |
| 54472 | TOLLIP | • | • |  | - | * | * |
| 57152 | SLURP1 |  | • |  |  |  |  |
| 80347 | COASY |  | • |  |  |  |  |
| 84617 | TUBB6 |  | • |  | - |  | * |
| 84676 | TRIM63 |  |  | • | - |  |  |
| 115650 | TNFRSF13C |  |  | • | - |  | * |
| 145389 | SLC38A6 |  |  |  | - |  |  |

Oblique bold letters represent the known disease genes.

• Genes verified by other methods;

- Genes existed in Cardiovascular GO Annotation Initiative Gene List;

* Genes shared the same annotations with known disease genes.
