# Supplementary material for: Predicting Candidate Genes Based on Combined Network Topological Features: A Case Study in Coronary Artery Disease
Source: PLoS One. 2012 Jun 22;7(6):e39542. doi: 10.1371/journal.pone.0039542 (PMC3382204; doi:10.1371/journal.pone.0039542)
Supplement: Table S2 — Pathway list of enrichment by known and candidate disease genes. (DOC) [file pone.0039542.s004.doc]

**Table S2. Pathway list of enrichment by known and candidate disease genes.**

| ***KEGG ID*** | ***Pathway Name*** | ***Known Disease Genes*** | ***Candidate Disease Genes*** | |
| --- | --- | --- | --- | --- |
| 04060 | Cytokine-cytokine receptor interaction | CX3CR1, IFNG, IL1A, IL1B, IL10, IL18, LTA, TNFRSF11B, CCL2, TNF, TNFRSF1A, CD40LG | IL3,TNFRSF13C,CCL2,TNFSF13B,IL1A,IL23A,TGFBR2,IL18R1,TNFRSF14,LTBR,CXCR4,TNFSF12,IL6ST,GHR,IL1R2,TNFRSF1A,TNFRSF12A,TNFRSF18,IL2RA,MET,IFNGR1,TNFRSF4,KIT,LTA,CCL11,PDGFRB, |  |
| 04810 | Regulation of actin cytoskeleton | F2, INS, ITGA2, ITGAM, ITGB3, RHOA, RAC1, MYLK2, CD14 | PPP1R12A,ITGA6,PIK3R1,MAP2K1,ITGA5,MYH9,CD14,MYL2,MAP2K2,CRKL,ITGA3,PIK3R5,ITGB3,PIK3R3,ACTB,ITGAV,MSN,F2R,PDGFRB, | |
| 04510 | Focal adhesion | CTNNB1, ITGA2, ITGB3, RHOA, RAC1, THBS4, MYLK2 | PPP1R12A,ITGA6,MAP2K1,ERBB2,MYL2,TNC,COL3A1,FLNB,CRKL,SHC1,ITGA3,IGF1R,PIK3R5,ITGB3,IGF1,PIK3R3,MET,PDGFRB,PDPK1,BIRC3,THBS2,PIK3R1,ITGA5,CAV1,ILK,GRB2,GSK3B,ACTB,COL1A2,ITGAV,COL4A6,MAPK10, | |
| 04010 | MAPK signaling pathway | IL1A, IL1B, NFKB1, RAC1, TNF, TNFRSF1A, CACNA1C, CD14 | HSPA8,MAP2K1,CD14,IL1A,TGFBR2,MAP2K2,MAPK8IP1,FLNB,MAP2K3,GRB2,CRKL,MAP3K7IP2,DAXX,MYC,IL1R2,TNFRSF1A,SRF,RELB,NFKB2,MAPK10,HSPB1,RELA,PDGFRB,MAP3K1, | |
| 04920 | Adipocytokine signaling pathway | IRS1, NFKB1, NPY, PRKAA1, TNF, TNFRSF1A, ADIPOQ, CD36 | IRS1,ACSL1,NFKBIE,RXRA,TNFRSF1A,PRKCQ,NFKBIB,PCK2,MAPK10,RELA,SOCS3, | |
| 04640 | Hematopoietic cell lineage | GP1BA, IL1A, IL1B, ITGA2, ITGAM, ITGB3, TNF, CD14, CD36, CD44 | IL3,CD55,ITGA6,ITGA5,CD14,IL1A,ANPEP,CD59,ITGA3,ITGB3,GYPA,IL1R2,CD9,IL2RA,CD44,KIT,MME, | |
| 04610 | Complement and coagulation cascades | F2, F12, FGB, CFH, SERPINE1, PLAU, PLAUR | CD59,SERPINE1,CD55,PLAU,F2R,PLAT,PLAUR, | |
| 04620 | Toll-like receptor signaling pathway | IL1B, NFKB1, RAC1, TLR4, TNF, CD14 | PIK3R1,MAP2K1,RIPK1,CD14,TOLLIP,MAP2K2,FADD,MAP2K3,TLR2,TLR7,TLR3,IRAK1,PIK3R5,MAP3K7IP2,STAT1,PIK3R3,LBP,MAPK10,RELA,TLR5, | |
| 04670 | Leukocyte transendothelial migration | CTNNB1, CYBA, GNAI2, ITGAM, RHOA, MMP2, MMP9, RAC1 | CXCR4,PIK3R5,PIK3R1,PIK3R3,ACTB,MYL2,MSN,GNAI1, | |
| 04080 | Neuroactive ligand-receptor interaction | LTB4R, AGTR1, AGTR2, EDN1, F2, NR3C1, NPY | EDNRA, F2R, F2RL1, GHR, LEPR, P2RY2 | |
| 04210 | Apoptosis | IL1A, IL1B, NFKB1, TNF, TNFRSF1A | IL3,IRAK1,BIRC3,PIK3R5,PIK3R1,TNFRSF1A,RIPK1,PIK3R3,CYCS,BCL2L1,IL1A,RELA,FADD | |
| 04020 | Calcium signaling pathway | AGTR1, NOS3, CACNA1C, MYLK2 | ERBB2,GNAQ,F2R,PDGFRB,NOS3,EDNRA | |
| 04910 | Insulin signaling pathway | GCK, INS, IRS1, PRKAA1 | PDPK1,TSC2,PIK3R1,SOCS2,PPP1R3A,MAP2K1,MAP2K2,SOCS3,GRB2,IRS1,CRKL,SHC1,PIK3R5,GSK3B,PIK3R3,PCK2,MAPK10, | |
| 03320 | PPAR signaling pathway | APOA5, FABP4, APOA1, APOA2, LPL, OLR1, PPARG, ADIPOQ, CD36 | PDPK1,FABP3,PPARD,ACSL1,RXRA,SLC27A4,LPL,ILK,PCK2,CYP27A1, | |
| 04512 | ECM-receptor interaction | GP1BA, ITGA2, ITGB3, THBS4, CD36, CD44 | ITGA3,THBS2,ITGA6,ITGB3,ITGA5,ITGAV,COL1A2,CD44,COL4A6,COL3A1,TNC, | |
| 04660 | T cell receptor signaling pathway | IFNG, IL10, RHOA, NFKB1, TNF, CD40LG | GRB2,PDCD1,NFKBIE,PIK3R5,PIK3R1,MAP2K1,GSK3B,PIK3R3,PRKCQ,NFKBIB,RELA,NFATC1,MAP2K2, | |
| 04930 | Type II diabetes mellitus | GCK, INS, IRS1, TNF, CACNA1C, ADIPOQ | IRS1,PRKCE,PIK3R5,PIK3R1,SOCS2,PIK3R3,MAPK10,SOCS3, | |
| 04310 | Wnt signaling pathway | CTNNB1, RHOA, LRP6, RAC1 | SMAD3,GSK3B,PPARD,MAPK10,FOSL1,NFATC1,MYC,AXIN2, | |
| 04520 | Adherens junction | CTNNB1, RHOA, RAC1 | ACP1,SMAD3,ERBB2,ACTB,MET,IGF1R,TGFBR2, | |
| 04630 | Jak-STAT signaling pathway | IFNG, IL10 | IL3,PIK3R1,SOCS2,BCL2L1,IL23A,SOCS3,GRB2,CISH,PIK3R5,IL6ST,MYC,GHR,STAT1,PIK3R3,IL2RA,IFNGR1,JAK3, | |
| 05220 | Chronic myeloid leukemia | NFKB1 | PIK3R1,MAP2K1,BCL2L1,TGFBR2,MAP2K2,CRKL,SMAD3,ABL1,GRB2,RB1,SHC1,PIK3R5,MYC,HDAC1,PIK3R3,CDKN1B,RELA, | |
| 05130 | Pathogenic Escherichia coli infection - EHEC | CTNNB1, RHOA, TLR4, CD14 | ABL1,ACTB,CD14,TUBB6,TLR5, | |
| 04940 | Type I diabetes mellitus | IFNG, IL1A, IL1B, INS, LTA, TNF | HSPD1,IL1A,LTA, | |
| 04514 | Cell adhesion molecules (CAMs) | ITGAM, SELE, SELP, CD40LG | PDCD1,ITGAV,CDH2,SELP,ITGA6,CD58, | |
| 04350 | TGF-beta signaling pathway | IFNG, RHOA, THBS4, TNF | SMAD3,BMP4,THBS2,TGFBR2,MYC,SP1, | |
| 04360 | Axon guidance | GNAI2, RHOA, RAC1 | ABL1,GSK3B,CXCR4,MET,NFATC1,GNAI1, | |
| 04650 | Natural killer cell mediated cytotoxicity | IFNG, RAC1, TNF | GRB2,SHC1,PIK3R5,PIK3R1,MAP2K1,PIK3R3,IFNGR1,NFATC1,MAP2K2, | |
| 04662 | B cell receptor signaling pathway | NFKB1, RAC1 | GRB2,NFKBIE,PIK3R5,PIK3R1,MAP2K1,GSK3B,PIK3R3,NFKBIB,NFATC1,RELA,MAP2K2, | |
| 04612 | Antigen processing and presentation | LTA | CALR,HSPA4,CTSB,HSP90AB1,HSPA5,HSPA8,B2M,HSP90AA1,CANX,LTA, | |
| 00480 | Glycerolipid metabolism | ALDH2, GLA, LIPC, LPL, LIPG | GSTP1,ANPEP | |
| 04530 | Tight junction | CTNNB1, GNAI2, RHOA | MYH9,PRKCE,PRKCQ,ACTB,MYL2,GNAI1, | |
| 05210 | Colorectal cancer | CTNNB1, RAC1 | GRB2,SMAD3,IGF1R,PIK3R5,MYC,PIK3R1,AXIN2,MAP2K1,GSK3B,PIK3R3,CYCS,MET,MAPK10,TGFBR2,PDGFRB, | |
| 04150 | mTOR signaling pathway | INS, PRKAA1 | PDPK1,PIK3R3,TSC2,HIF1A,PIK3R5,PIK3R1,IGF1, | |
| 05212 | Pancreatic cancer | NFKB1, RAC1 | SMAD3,RB1,PIK3R5,PIK3R1,STAT1,MAP2K1,ERBB2,PIK3R3,BCL2L1,MAPK10,TGFBR2,RELA | |
| 04370 | VEGF signaling pathway | NOS3, RAC1 | PIK3R3,PIK3R5,HSPB1,NFATC1,MAP2K2,PIK3R1,MAP2K1,NOS3, | |
| 05010 | Alzheimer's disease | LPL | IDE,APBB1,TNFRSF1A,PSEN2,GSK3B,CYCS,GNAQ,LPL,APP,FADD,MME, | |
| 00010 | Glycolysis / Gluconeogenesis | ALDH2, GCK, LDHB | PGM1,GPI,PCK2, | |
| 05120 | Epithelial cell signaling in Helicobacter pylori infection | NFKB1, RAC1 | MET,MAPK10,RELA, | |
| 04912 | GnRH signaling pathway | MMP2, CACNA1C | GRB2,PLD2,GNAQ,MAPK10,MAP2K2,MAP2K3,MAP3K1,MAP2K1, | |
| 04950 | Maturity onset diabetes of the young | GCK, INS | NR5A2,HNF4A | |
| 04664 | Fc epsilon RI signaling pathway | RAC1, TNF | GRB2,PRKCE,IL3,PIK3R5,PIK3R1,MAP2K1,PIK3R3,MAPK10,MAP2K2,MAP2K3,, | |
| 04730 | Long-term depression | GNAI2, NOS3 | GNAQ,IGF1R,MAP2K2,MAP2K1,GNAI1,IGF1, | |
| 00350 | Tyrosine metabolism | MIF, TH | MIF,COMT | |
| 04540 | Gap junction | GNAI2 | GRB2,GNAQ,TUBB6,DRD2,MAP2K2,PDGFRB,MAP2K1,GNAI1, | |
| 04720 | Long-term potentiation | CACNA1C | GNAQ,PPP1R12A,MAP2K2,MAP2K1, | |
| 00120 | Bile acid biosynthesis | ALDH2 | CYP27A1,CYP46A1 | |
| 00561 | Glycerophospholipid metabolism | LCAT | LPL,PPAP2A,AGPAT2,MGLL, | |
| 05020 | Parkinson's disease | TH | IL1A,MAP2K2,HSPA5,MAP2K1,PRNP, | |
